# Comparative study of some non-Newtonian nanofluid models across stretching sheet: a case of linear radiation and activation energy effects

**DOI:** 10.1038/s41598-024-54398-x

**Published:** 2024-02-28

**Authors:** Syed Asif Ali Shah, Muhammad Idrees, Abdul Bariq, Bilal Ahmad, Bagh Ali, Adham E. Ragab, Emad A. Az-Zo’bi

**Affiliations:** 1 Department of Mathematics and Statistics, The University of Lahore, Lahore, Pakistan; 2 Department of Mathematics, Laghman University, Mehtarlam City, Laghman 2701 Afghanistan; 3 School of Mechanical Engineering and Automation, Harbin Institute of Technology, Shenzhen, 518055 China; 4 Department of Industrial Engineering, College of Engineering, King Saud University, P.O. Box 800, 11 421 Riyadh, Saudi Arabia; 5 Department of Mathematics, Mutah University, Mutah, Al Karak, Jordan

**Keywords:** Nanofluids, Non-Newtonian fluid models, Casson nanofluid, Williamson nanofluid, Prandtl nanofluid, Thermal radiations, Activation energy, Engineering, Mathematics and computing, Nanoscience and technology, Physics

## Abstract

The use of renewable energy sources is leading the charge to solve the world’s energy problems, and non-Newtonian nanofluid dynamics play a significant role in applications such as expanding solar sheets, which are examined in this paper, along with the impacts of activation energy and solar radiation. We solve physical flow issues using partial differential equations and models like Casson, Williamson, and Prandtl. To get numerical solutions, we first apply a transformation to make these equations ordinary differential equations, and then we use the MATLAB-integrated bvp4c methodology. Through the examination of dimensionless velocity, concentration, and temperature functions under varied parameters, our work explores the physical properties of nanofluids. In addition to numerical and tabular studies of the skin friction coefficient, Sherwood number, and local Nusselt number, important components of the flow field are graphically shown and analyzed. Consistent with previous research, this work adds important new information to the continuing conversation in this area. Through the examination of dimensionless velocity, concentration, and temperature functions under varied parameters, our work explores the physical properties of nanofluids. Comparing the Casson nanofluid to the Williamson and Prandtl nanofluids, it is found that the former has a lower velocity. Compared to Casson and Williamson nanofluid, Prandtl nanofluid advanced in heat flux more quickly. The transfer of heat rates are $25.87\%$, $33.61\%$ and $40.52\%$ at $Rd =0.5, Rd=1.0$, and $Rd=1.5$, respectively. The heat transfer rate is increased by $6.91\%$ as the value of *Rd* rises from 1.0 to 1.5. This study is further strengthened by a comparative analysis with previous research, which is complemented by an extensive table of comparisons for a full evaluation.

## Introduction

During the American Society of Mechanical Engineers annual conference in 1995, Choi presented a term called “nanofluid” to describe a distinctive category of heat-transfer fluids that utilize nanotechnology and exhibit superior thermal properties compared to their base fluids or conventional particle-fluid suspensions^1^. A nanofluid is composed of nanoparticles, which are characterized by their sub-nanometer size. Fluids are transported via a suspension of fluids in colloidal form. Nanoparticles within nanofluidic systems are derived from a variety of sources, including metals, oxides, carbides, and carbon nanotubes. According to^2^, nanofluids possess an inherent ability to retain significant particles within their plane. The utilization of nanoparticles has been found to improve the thermophysical properties and heat transfer characteristics. The predominant area of interest in nanofluids research pertains to the application of surface science and colloidal theory to materials. The characteristics of nanofluids have been extensively studied by researchers who have dispersed various types of nanomaterials in different proportions. The physicochemical properties of nanofluids exhibit significant variations from their solid counterparts, owing to their immense importance in nanobiomedical technologies^3^.

The heat transmission capacity of a material is influenced by its composition and structure. The study of the utilization of nanofluids involves both empirical and theoretical methodologies. Nanoparticles have been implemented in the cooling mechanisms of various electrical apparatuses. Nanofluids utilize Brownian diffusion as a slip mechanism. The temporal behavior of a non-Newtonian fluid is contingent upon its specific characteristics, which may render it either time-invariant or time-variant. Nanofluids have been implemented in engineering applications as a substitute for conventional heat transfer fluids to enhance the energy efficiency of systems^4^. Using nanofluids on a linear surface to generate boundary flows presents several benefits for engineering and industrial purposes. According to^5^, nanofluids can improve thermal efficiency, acceleration of processes, and extension of equipment lifespan. The structural characteristics of nanoparticles, which resemble those of solids, promote the transmission of nanofluids. The significance of nanofluid lies in its potential applications, including but not limited to heat transfer enhancement, energy conversion and storage, biomedical engineering, advanced manufacturing, coatings, energy efficiency in buildings, aerospace industries, and environmental and sustainable technologies. Despite ongoing research and development, a comprehensive understanding of nanofluids’ behavior, stability, and long-term effects remains a research subject. The performance of solar cells was improved by Sheikholeslami and Khalili^6^ using a cooling technique that used nanofluid jet interaction. Acharya^7^ investigated the hybrid nanofluidic transfer of $MWCNT-Fe_3O_4$-water via a micro-wavy tube. Sheikholeslami and Khalili used nanofluid filtration to increase a concentrated solar heating (CPVT) module’s performance.^8^

The Williamson fluid model is a notable model for non-Newtonian fluids, particularly pseudo-plastic fluids, due to its incorporation of both maximum and minimum viscosities^9^. In^10^, the viability of thorough analysis for a three-dimensional Williamson nanofluid flow under Darcy–Forchheimer conditions over a stretched surface is examined. Yahya et al.^11^ mathematically simulated Williamson nanofluid intensity flow under MHD and heat radiation. Bioconvection and Cattaneo–Christov thermal motion affected nanofluid transport across an expanding surface with a convective limit^12^. Shafiq and Sindhu hypothesized and investigated radiation-driven Williamson flow^13^. Sreedevi and Reddy examined the hybridization of a nanofluid stream with a Cattaneo-Christov heat fluid tactic microorganism across a revolving plate^14^. A two-dimensional second-grade magnetohydrodynamic fluid flow approaching a porous rapidly stretched surface with heterogeneous-homogeneous responses was studied by Khan et al.^15^. Sheikholeslami and Jafaryar^16^ assessed the turbulence of an oil-based hybrid nanofluid in the absorbing pipe of a concentrated solar system.

The Casson nanofluid is a burgeoning field of study that amalgamates the tenets of nanofluidics and the Casson fluid model to scrutinize the rheological characteristics of fluid suspensions that encompass nanoparticles. Nanofluids comprising a host fluid and dispersed nanoparticles demonstrate superior thermal and mechanical characteristics compared to conventional fluids. The unstable motion of a non-Newtonian Casson nanofluid was studied by Jamshed et al.^17^ in terms of entropy and heat transfer. Using heat transfer of Cassona transverse the linearly extending sheet, Tawade et al.^18^ investigated the problem of constant laminar flow of nanofluid on a two dimensional boundary layer.

The discipline of Prandtl nanofluids is an emerging area of study that combines the concepts of Prandtl boundary layer theory and nanofluidics to study the thermal properties of fluid suspensions containing nanoparticles. Acharya^19^ investigated the effects of nanoparticles size and the solid-liquid interface layer on the unstable ferrous-water nanoliquid flow on a rotating disc. The electro-osmotic flow of Prandtl nanofluids was investigated by Abbasi et al.^20^ using Keller box simulations while considering thermal and solutal slip flow constraints. The mathematical study of a magnetised nanofluidic channel within a cubic-shaped cage with an interior circle cylinder installed inside the cube was conducted by Acharya^21^. The cross-diffusion implications of a conduction heated stretched plate on radiation and reacting Prandtl nanofluid were studied by Patil et al.^22^.

The study of magnetohydrodynamics significantly influences the field of fluid dynamics. The phenomenon of MHD heat and mass transmission across an extended surface finds practical applications in various fields such as metallurgy, polymer chemistry, and glass fiber manufacturing^23^. The research conducted by Shahzadi and Nadeem pertained to the analysis of inclined magnetic fields in relation to metallic nanoparticles that are submerged in blood and subject to convective boundary conditions (BCs)^24^. Various disciplines, including electrochemistry, chemical engineering, geophysics, astronomy, and polymer processing, have extensive uses for the behavior of MHD laminar boundary layer flow across a stretched surface^25^. Haider et al.^26^ examined the impact of fluid flow and a stretched sheet on the MHD stagnation factor. Lund et al.^27^ proposed a triple solution approach that accounts for the effects of Joule heating and viscous dissipation beyond an exponentially decreasing sheet. The impact of a radial magnetic field on the stimulation of metallic nanoparticles through eccentric cylinders was investigated by Shahzadi and Nadeem^28^. Different authors have also discussed MHD fluid’s heat transfer rate across various geometries^29,30^.

Thermal radiation refers to the emission of electromagnetic radiation that arises from the thermal agitation of particles. Radiation is formed when heat from the movement of protons and electrons (in common types of material) inside the fabric is turned into electromagnetic radiation. Each residing material emits energy continually owing to its temperature^31^. This kind of energy is referred to as thermal radiation. Extremely hot bodies alternate thermal strength until they attain a temperature comparable to their surroundings. Radiation and temperature are inextricably linked. Radiative heat flow, essential in industrial sectors, impacts environmental and commercial activities^32^. The study conducted by^33^ examined the impact of buoyancy on MHD slip heat transfer during the flow of a nanofluid over a vertical porous plate. The effects of Stefan blowing on an unstable nanofluid MHD float on a stretched sheet were examined by^34^ using electrically driven proximity. The study conducted by Asjad and colleagues investigated the effects of bio-convection and chemical reaction on the MHD nanofluid drift over an exponentially expanding sheet^35^. Researchers^36^ have examined Prandtl nanofluid drift in porous media convective systems using heat radiation and higher-order chemical processes.

Activation energy pertains to the minimal quantity of electrical energy that is necessary for a substance to instigate particular chemical reactions and transactions. Swante Arrhenius introduced the concept of activation energy in 1889^37^. In order for a chemical reaction to occur, it is necessary for the reactants to possess a specific quantity of energy, referred to as activation energy. The quality of potential issues or strength is a determining factor in separating materials and potential strength devices at their respective minimums. The aforementioned methodology demonstrates a heterogeneous array of household appliances across multiple domains, including but not limited to industrial engineering, oil storage, geothermal production, base liquid mechanics, oil emulsification, and food preparation^38^. The phenomenon of boundary layer gliding of heat and mass motion can be explained through the principles of electricity. This process involves the operation of the boundary divider on its ground, which can be represented mathematically as a dual-substance reaction via an Arrhenius actuator^39^. The theoretical importance of temperature-dependent viscosity and partial slippage in the biological convection evaluation of a Maxwell nanofluid contained by an expanded surface was discussed by Khan et al.^40^.

It is noted that there need to be more studies on the comparative analysis of non-Newtonian fluid models in the literature, as mentioned above. Furthermore, more discourse should be on the impact of heat radiation, activation energy, and non-Newtonian nanofluids on the stretching sheet phenomenon. The present study aims to determine the heat transfer phenomenon in various non-Newtonian nanofluid models, including Williamson, Casson, and Prandtle. Furthermore, the assessment encompasses the determination of activation energy and the analysis of thermal radiation outcomes. The mathematical conundrum is expressed via a set of partial differential equations (PDEs), which are addressed by means of a similarity transformation that converts the PDEs into ordinary differential equations (ODEs). The MATLAB built-in function BVP4C is utilized to derive the numerical solution. Subsequently, an analysis is conducted on the graphical patterns exhibited by the important parameters. Graphs and tabular data are utilised to clarify secondary flow characteristics like skin friction coefficient and heat transfer rate. The following research questions are the focus of this article:

- What is the behaviour of various non-Newtonian nanofluid models on stretched sheets?
- What effect does linear radiation have on the behaviour of heat transport in these models of nanofluids?
- What is the impact of activation energy implications for the thermal properties of non-Newtonian nanofluids on streached sheets?
- To what extent can one use the study findings to apply in everyday and practical scenarios?
- What parallels and divergences exist between the results of the present study and those from earlier investigations?

## Mathematical formulation

**Figure 1:**
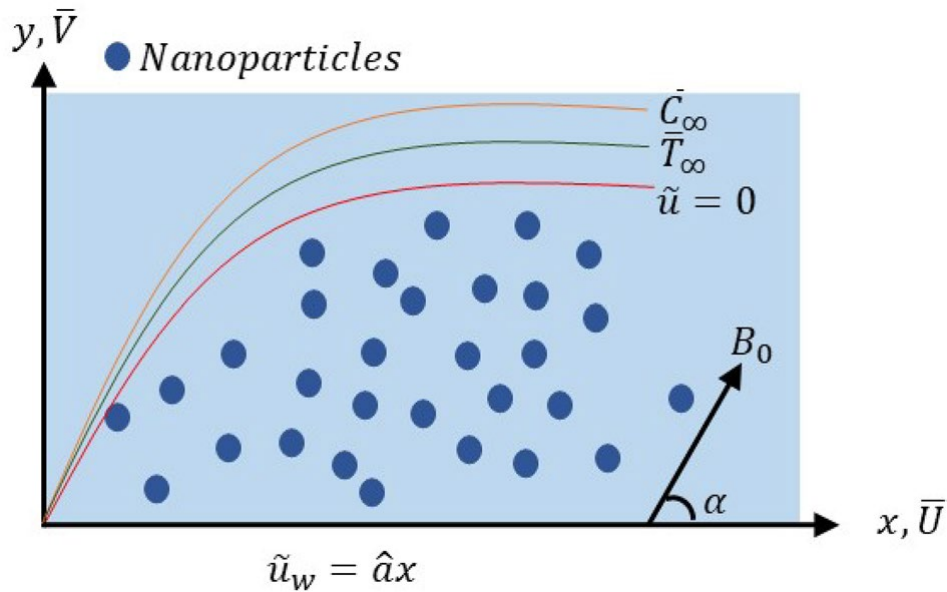
Problem structure.

Suppose a two-dimensional uniform flow across a stretching sheet along $y>0$. Applying the magnetic field perpendicularly to the flow and making an angle $\alpha$ with the *x*-axis. Consider the physical attributes represented at surface concentration and temperature, which are $\bar{T}_{w}$, $\bar{C}_{w}$, $\bar{T}_{\infty }$, $\bar{C}_{\infty }$. Also, consider that the velocity of the stretching sheet is $\grave{u_{w}}(x) = \hat{a}x$, where $\hat{a}$ is a positive constant. The coordinates and flow mode are illustrated in Fig. 1. The Cauchy stress tensor for the models in discussion is defined as:

$$\begin{aligned} \bar{S}=\bar{\tau }_{1}-\bar{p}\bar{I}, \end{aligned}$$

with $\bar{\tau }_{1}$ a stress tensor.

$$\begin{aligned} \bar{S}=-\bar{P}\bar{I}=\hat{A}_{1}\left( \frac{\grave{\mu }_{0}-\grave{\mu }_{\infty }}{1-\check{\Gamma }\bar{\dot{\gamma }}+\grave{\mu }_{\infty }}\right) , \end{aligned}$$

where $\grave{\mu }_{\infty }$ is the shear rate of infinity, $\grave{\mu }_{0}$ stands for the limiting viscosity at zero, $\hat{A}_{1}$ indicates the 1st Rivlin–Ericksen tensor, and $\check{\Gamma }>0$ denotes the time constant. Moreover, it can be written as^41,42^

$$\begin{aligned} \bar{\dot{\gamma }}=\sqrt{\frac{\pi }{2}},\pi =trace(\hat{A}_{1}^{2}). \end{aligned}$$

1 $$\begin{aligned} \bar{\dot{\gamma }}=\sqrt{\left[ \left( \frac{\partial \bar{U}}{\partial x}\right) ^{2}+\frac{1}{2}\left( \frac{\partial \bar{U}}{\partial y}+\frac{\partial \bar{V}}{\partial x}\right) ^{2} + \left( \frac{\partial \bar{V}}{\partial y}\right) ^{2} \right] }. \end{aligned}$$

Suppose only the case of $\grave{\mu }_{\infty }=0$ and $\check{\Gamma }\bar{\dot{\gamma }}<1$, so we contain^43^

$$\begin{aligned} \bar{\tau }_{1}=\left[ \hat{A}_{1}\left( (1+\check{\Gamma }\bar{\dot{\gamma }})\right) ^{-1} \grave{\mu }_{0}\right] , \end{aligned}$$

or

2 $$\begin{aligned} \bar{\tau }_{1}=\left[ \hat{A}_{1}\grave{\mu }_{0}\left( 1+\check{\Gamma }\bar{\dot{\gamma }}\right) \right] . \end{aligned}$$

According to the above assumptions, the governing equations of the non-Newtonian fluid models are shown below^44–47^:

3 $$\begin{aligned}{}&\frac{\partial \bar{U}}{\partial x}+\frac{\partial \bar{V}}{\partial y} =0, \end{aligned}$$

4 $$\begin{aligned}{}&\bar{U}\frac{\partial \bar{U}}{\partial x}+\bar{V}\frac{\partial \bar{U}}{\partial y}=\nu \frac{\partial ^2 \bar{U}}{\partial y^2}+\sqrt{2}\check{\Gamma }\nu \frac{\partial \bar{U}}{\partial y}\dfrac{\partial ^2 \bar{U}}{\partial y^2}-\frac{\sigma B_{0}^{2}\bar{U}}{\rho _{f}}\sin ^{2}(\alpha )\nonumber \\&+\nu \left( 1+\frac{1}{B}\right) \frac{\partial ^2\bar{U}}{\partial y^2}+\frac{\hat{A}}{\rho \check{C_{1}}} \frac{\partial ^2\bar{U}}{\partial y^2}+\frac{\hat{A}}{2\rho \check{C_{1}^{3}}} \left( \frac{\partial \bar{U}}{\partial y}\right) ^2 \frac{\partial ^2\bar{U}}{\partial y^2} , \end{aligned}$$

5 $$\begin{aligned}{}&\bar{U}\frac{\partial \bar{T}}{\partial x}+\bar{V}\frac{\partial \bar{T}}{\partial y}=\breve{\alpha } \frac{\partial ^2\bar{T}}{\partial y^2}+\bar{\tau }\left[ \left( \frac{\partial \bar{T}}{\partial y}\right) ^{2} \frac{\tilde{D}_{T}}{\bar{T}_{\infty }}+\frac{\partial \bar{T}}{\partial y}\tilde{D}_{B} \frac{\partial \bar{C}}{\partial y}\right] +\frac{1}{(c\rho )_{nf}}\left( \frac{\partial \hat{q}^{**}_{r}}{\partial y}\right) , \end{aligned}$$

6 $$\begin{aligned}{}&\bar{V}\frac{\partial \bar{C}}{\partial y}+\bar{U}\frac{\partial \bar{C}}{\partial x} =\frac{\partial ^2 \bar{T}}{\partial y^2}\tilde{D_{B}}+\left( \frac{\partial ^2\bar{T}}{\partial y^2}\right) \frac{\tilde{D_{T}}}{\bar{T}_{\infty }}-k_{r}^2(\bar{C}-\bar{C}_{\infty }) \left( \frac{\bar{T}}{\bar{T_{\infty }}}\right) ^n \exp \left( \frac{-Ea}{\bar{T}\bar{k}}\right) . \end{aligned}$$

The boundaries are given by

7 $$\begin{aligned}{}&\text {at} ~y=0 ~\bar{U}=\grave{u}_{w}\left( x\right) =\hat{a}x, \bar{V}=0, \frac{\partial \bar{T}}{\partial y}=(\bar{T}-\bar{T}_{f})\frac{\bar{h}}{\bar{ k}}, \bar{C}_{w}=\bar{C} , \end{aligned}$$

8 $$\begin{aligned}{}&\text {at}~y\rightarrow \infty ~\bar{U}=0,\,\,\,\bar{C} =\bar{C}_{\infty },\,\,\, \bar{T} =\bar{T}_{\infty }. \end{aligned}$$

The similarity transformations are given by^48–50^

9 $$\begin{aligned} \eta =y \sqrt{\frac{\hat{a}}{\nu }} ,\theta (\eta )=\frac{\bar{T}-\bar{T}_{\infty }}{\bar{T}_{f}-\bar{T}_{\infty }}, \phi (\eta )=\frac{\bar{C}_{\infty }-\bar{C}}{\bar{C}_{w}-\bar{C}_{\infty }}, \hat{\psi }=f(\eta )\sqrt{\hat{a}\nu }x, \end{aligned}$$

where the stream function $\hat{\psi }$ is defined as

$$\begin{aligned} \bar{U}=\frac{\partial \hat{\psi }}{\partial y}, ~\bar{V}=-\frac{\partial \hat{\psi }}{\partial x}. \end{aligned}$$

The controlling PDEs (4), (5), and (6) are transformed into ODEs, as shown below, after performing the similarity transformation.

10 $$\begin{aligned}{}&f'''(1+f''\hat{\lambda }_{1})+ ff''-f'(M\sin ^{2}\gamma +f')+\left( 1+\frac{1}{\breve{B}}\right) f'''+\left[ \epsilon +\acute{\delta } f''^2\right] f''' =0, \end{aligned}$$

11 $$\begin{aligned}{}&\frac{1}{Pr}\left( 1+\frac{4}{3}Rd\right) \theta ''+\theta '\phi 'Nb+f\theta '+\theta '^{2}Nt =0, \end{aligned}$$

12 $$\begin{aligned}{}&\phi ''+fLe\phi '+\frac{Nt}{Nb}\theta ''-Le \tilde{\delta }_{c}\left[ 1+\theta \hat{\delta }\right] ^n \exp \left( \frac{-E}{1+ \theta }\hat{\delta }\right) \phi =0, \end{aligned}$$

with boundary conditions

13 $$\begin{aligned}{}&f=0,\,\,\,\phi =1,\,\,\, f'=1, \,\,\, \theta '=Bi(\theta -1), \,\,\, \text {at} ~\eta =0, \end{aligned}$$

14 $$\begin{aligned}{}&f'\rightarrow 0, \,\,\, \phi \rightarrow 0, \,\,\, \theta \rightarrow 0,\,\,\, \text {at}~\eta \rightarrow \infty , \end{aligned}$$

where $\hat{\lambda }_{1}=\sqrt{\frac{2\hat{a}}{\nu }}\hat{a}x\check{\Gamma }$ is Williamson parameter, $Nt=\frac{\bar{\tau }\tilde{D}_{T}\left( \bar{T}_{f}-\bar{T}_{\infty }\right) }{\bar{T_{\infty }\nu }}$ denotes Thermophoresis parameter, , $Pr=\frac{\nu }{\breve{\alpha }}$ shows the Prandtl number, $Rd=\frac{4\sigma ^{*}T_{\infty }^{3}}{K^{*}k}$ represents the radiation number, $Nb=\frac{\tau _{1}D_{B}(C_{w}-C_{\infty })}{\nu }$ , $Bi=\frac{\bar{h}}{\bar{k}}\sqrt{\frac{\nu }{\hat{a}}}$ is Biot number, $Le=\frac{\nu }{\tilde{D}_{B}}$ signifies the Lewis number, $\acute{\delta }=\frac{\hat{a}^{3}x^{2}\hat{A}}{2\bar{\nu _{f}}C_{1}^{3}}$, $\epsilon =\frac{\hat{A}}{C_{1}}$ denotes the fluid parameters, magnetic parameter is specified by $\check{M}=\frac{\sigma B_{0}^{2}}{\rho _{f}\hat{a}}$, $E = \frac{Ea}{\bar{k}\bar{T}}$, $\hat{\delta }=\frac{\bar{T}_{w}-\bar{T}_{\infty }}{\bar{T}_{\infty }}$ defined the activation energy, $Nb=\frac{\bar{\tau }\tilde{D}_{B}\left( \bar{C}_{w}-\bar{C}_{\infty }\right) }{\nu }$ defines the Brownian motion parameter and $\tilde{\delta }_{c}=\frac{Kr^2}{\hat{a}}$ be the chemical reaction parameter.

The physical parameters of concern the momentum like that, skin-friction coefficient $(\hat{C}_{fx})$, local Nusselt number $(\hat{N}u_{x})$, local Sherwood number $(\hat{S}h_{x})$, respectively. The parameters described as

15 $$\begin{aligned} \hat{C}f_{x}=\frac{\acute{\tau }_{w}}{\rho \grave{u}^{2}_{w}}, \hat{N}u_{x}=\frac{x\check{q}_{w}}{\bar{T}_{w}-\bar{T}_{\infty }}, \hat{S}h_{x}=\frac{x\check{q}_{m}}{\tilde{D}_{B}(\bar{C}_{w}-\bar{C}_{\infty })}. \end{aligned}$$

At the surface, $\acute{\tau }_{w}=\mu \left[ \left( \frac{\hat{A}}{ \check{C_{1}}}+\frac{\check{\Gamma }}{\sqrt{2}}\frac{\partial \bar{U}}{\partial y}+\frac{\hat{A}}{2 \check{C_{1}^{3}}}(\frac{\partial \bar{U}}{\partial y})^{2}\right) \frac{\partial \bar{U}}{\partial y}\right] |_{y=0}$ is shear stress, $\check{q}_{w}=-(\frac{\partial \bar{T}}{\partial y}+\frac{4\sigma ^{*}T_{\infty }^{3}}{K^{*}k}\frac{\partial \bar{T}}{\partial y})_{y=0}$ is heat flux, $\check{q}_{m}=-\tilde{D}_{B}(\frac{\partial \bar{C}}{\partial y})_{y=0}$ is mass flux. By solving these quantities along similarity transformation, we get

$$\begin{aligned} \hat{C}f_{x}=Re_{x}^{-0.5}\left( 1+\frac{1}{\breve{B}}\right) \left( \epsilon +\frac{\hat{\lambda }_{1}}{2}f''(0)+\acute{\delta }(f''(0))^{2}\right) f''(0),\\ {\hat{{N}}u_{x}=-Re_{x}^{0.5}(1+\frac{4}{3}Rd)\theta '(0)},\\ \hat{{S}h}_{x}=-Re_{x}^{0.5}\phi '(0), \end{aligned}$$

where, $Re_{x}=\frac{\grave{u}_{w}x}{\nu }$ is a Reynolds number.

## Numerical methodology

In the field of Engineering, control theory, applied mathematics, theoretical physics, and optimization all deal with such problems heaving boundary value conditions. Since it is frequently difficult to solve boundary-value conditions analytically that is the reason numerical methods must be applied. These boundary value conditions-based problems are commonly solved numerically using shooting techniques. The circumstance required minimum planning and must be easy for shooting tactics. Shooting techniques are beneficial for solving nonlinear differential equations because they offer a variety of benefits to the problem solver. A large range of differential equations may be solved using a number of shooting approaches, which are extremely adaptable. It is impossible to solve the coupled DEs (10–12) with boundary assumptions (7, 8) analytically due to their significant nonlinearity. The MATLAB application is used to tackle nonlinear coupled boundary problems using various numerical techniques. In comparison with previous numerical techniques, shooting technique is more accurate and has a $10^{-6}$ tolerance^51^. Since bvp4c is available in widely used numerical computing tools such as MATLAB, and since it is resilient, accurate, efficient, and adaptable, it is a preferable solution for boundary value issues for ODEs. Engineers and researchers in a variety of scientific and technical domains use it because of its versatility in handling a large number of problems-including those involving nonlinearities and mixed boundary conditions-and its capacity to produce precise answers with a low computer resource need. In order to translate coupled DEs (9-12) into first-order ODEs, the following notations are used: $f=y_{1},~ f'=y_{2},~ f''=y_{3},~ f'''=yy1,~ \theta =y_{4},~ \theta '=y_{5},~ \theta ''=yy2,~ \phi =y_{6},~ \phi '=y_{7},~ \phi ''=yy3$

16 $$\begin{aligned}{}&yy1=\frac{1}{(1+\hat{\lambda } y_{3})+(1+\frac{1}{\breve{B}})+\epsilon (1+\acute{\delta } y_{3}^2)} [-y_{1} y_{3}+y_{2}(Msin\gamma +y_{2})], \end{aligned}$$

17 $$\begin{aligned}{}&yy2= - \frac{Pr}{1+\frac{4}{3}Rd} \left[ -Nt y_{5}^{2} - Nb y_{5} y_{7} - y_{5} y_{1}\right] , \end{aligned}$$

18 $$\begin{aligned}{}&yy3= \frac{Nt}{Nb}yy2 - Le y_{1}y_{7}+ Le \delta _{c} \left[ 1+y_{4}\hat{\delta }\right] ^n \exp \left( \frac{-E}{1+y_{4}\hat{\delta }}\right) y_{6}. \end{aligned}$$

along with BCs:

19 $$\begin{aligned}{}&y_{1}=0, y_{5}=Bi(1-y_{4}), y_{2}=1, y_{6}=1, y_{7}=1 ~\text {at} ~\eta =0, \end{aligned}$$

20 $$\begin{aligned}{}&y_{2}\rightarrow 0, y_{4}\rightarrow 0, y_{6}\rightarrow 0 ~\text {at}~\eta \rightarrow \infty . \end{aligned}$$

### Code verification

To validate our latest findings, we examined the skin friction and Nusselt number values with the values from Khan and Pop^52^ and Srinivasulu and Goud^2^, assuming $Pr=6.2$ and setting all other parameters to zero. There is a high degree of agreement with the body of current literature (See Table 1).

**Table 1:** Camparison of Nusselt number for different values of *Pr* while $Bi \rightarrow \infty$ and all other parameters equal zero.

$P_{r}$	Khan and Pop^52^	Srinivasulu and Goud^2^	Present outcomes
0.2	0.1691	0.1732	0.172349
0.7	0.4539	0.4539	1.453917
2.0	0.4113	0.9113	0.911361
7.0	1.8954	1.8954	1.395431
20.0	3.3539	3.3539	3.353549
70.0	6.4621	6.4621	6.462249

## Results and discussion

This section presents a comparison of the temperature, velocity, and concentration profiles of the Prandtl, Casson, and Williamson nanofluid models.

**Figure 2:**
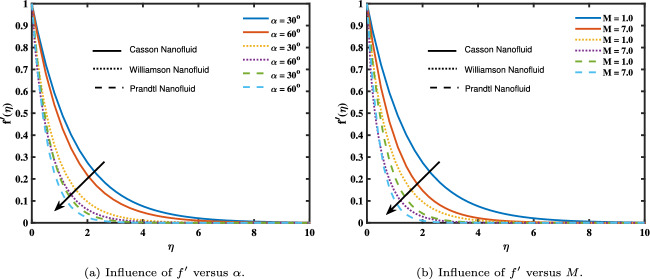
Influence of angle of inclination and magnetic parameter on velocity.

Each model is calculated numerically by giving fixed inputs to parameters such as $1\le M$, $\le 7.0$, $30^\circ \le \alpha \le 60^\circ$, $0.3\le Nb\le 1.5$, $0.2\le Nt\le 0.7$, $-0.03\le QT\le -0.1$, $0.2\le QE\le o.6$, $0.1\le Le\le 0.54$, and $0.5\le \sigma \le 1.5$. The influence of magnetic effects (*M*) and the ($\alpha$) inclination on velocity profiles is depicted in Fig. 2a and b. As ($\alpha$) rises, the fluid’s velocity rates decrease. The study’s findings suggest that at large turn and magnetic parameter levels, speeds drop when magnetic fields are applied. As (*M*) and ($\alpha$) increase, a force from the Lorentz forces causes the fluid to undergo a slowing of its velocity. This phenomenon is caused by the interaction of the electrically conductive fluid and the flowing magnetic field, which produces a strong resistive force known as the Lorentz force^34^.

**Figure 3:**
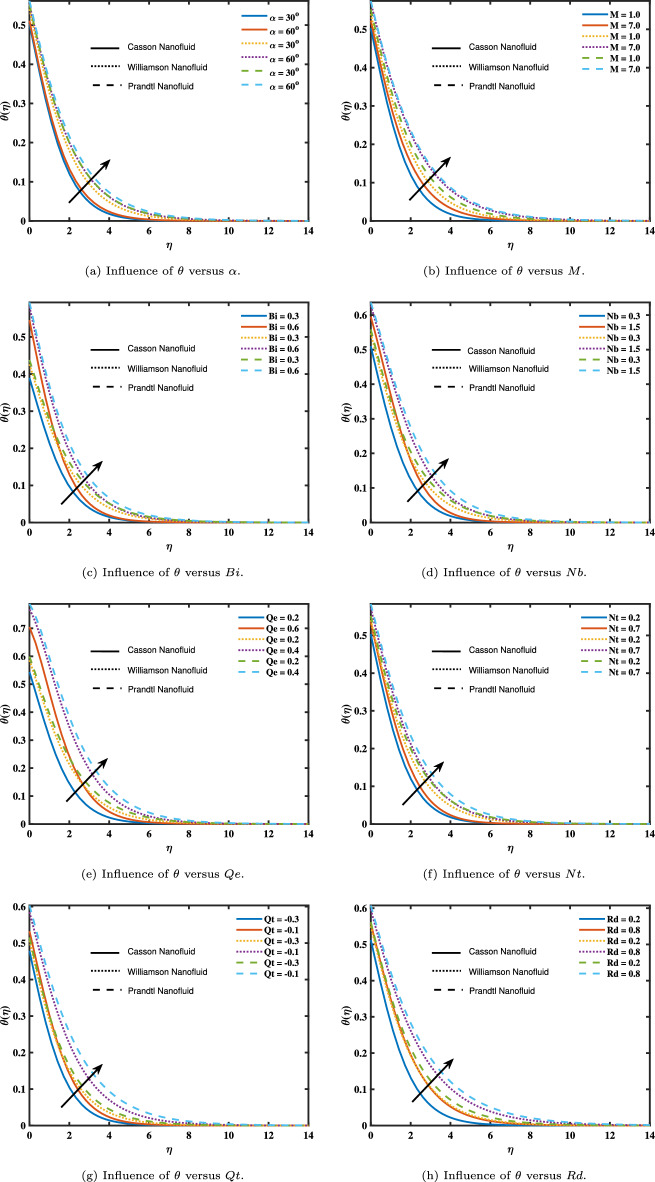
Impact of angle of inclination, magnetic parameter, Biot number, thermophoresis parameter, Brownian motion parameter, source/sink parameters, and thermal radiation on temperature.

Figure 3 depicts the dimensionless temperature variations ($\theta (\eta )$) for comparing nanofluid models connected to the various flow parameters. The influence of (*M*) and ($\alpha$) on temperature profiles are seen in Fig. 3a and b. The temperature grows as inputs of ($\alpha$) and (*M*) rise. Figure 3c and h illustrate the impact of (*Rd*) and (*Bi*) on temperature. As a result of exponential and thermal heat source-sink relationships, Fig. 3e and g show an increase in temperature profiles. The nonlinear thermal radiation variable and the ratio temperature variable both have higher values, increasing and boosting heat transfer inside the fluid. There are three key ramifications of the nonlinear thermal radiation parameter’s favorable influence on the nanofluid’s temperature. First, the boundary layer’s temperature rises, and all heat transmission distances are maintained uniformly inside the layer. Second, the nanoparticles inside the fluid gain thermal energy because of their thermal conductivity, which increases diffusion and heat transmission. Third, Thermoconduction through load and other nanofluid thermal transfer methods are optimized. The temperature dependency of (*Nt*) and (*Nb*) can be seen in Fig. 3f and d. As the amount of these factors rises, the temperature rises. This is so that the liquid temperature within the boundary layer can be raised by the thermophoresis force, which pulls heated particles from the hot surface into the cold zone. These elements physically specify the thermal boundary layer. As a result, as (*Nt*) and (*Nb*) increase, the temperature rises.

**Figure 4:**
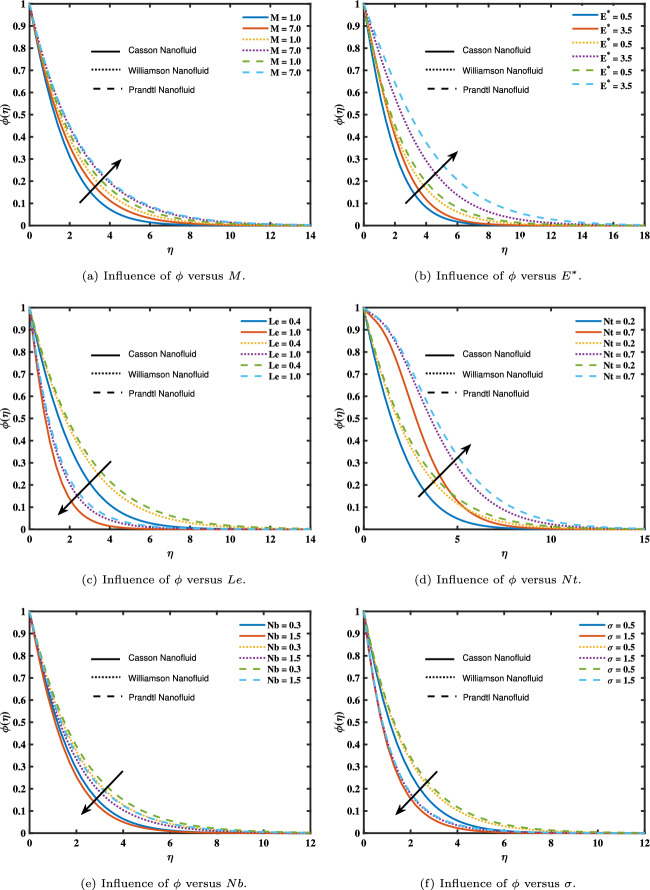
Influence of magnetic parameter, activation energy parameter, Lewis number, thermophoresis parameter, Brownian motion parameter, and chemical reaction on concentration.

The relationship between many essential parameters and the concentration variation ($\phi$) is shown in Fig. 4. Figure 4a and c show how (*M*) and (*Le*) affect the concentration curves. The concentration rises with increasing (*M*) values but falls with increasing (*Le*) values. This effect is caused by the high level of the Lewis number; a high value diminishes the thickness of the concentration boundary layer. Consequently, when (*Le*) rises, concentration falls. Figure 4d and e show how (*Nt*) and (*Nb*) respond to the concentration curves. The concentration rises with large values of (*Nt*) but drops with larger values of (*Nb*). The concentration field expanded physically as (*Nt*) grew because nanoparticles moved to a cooler region as it grew. The fluid’s temperature difference and shear gradient are represented by the variable Nt. An increasing temperature differential and shear gradient over the boundary layer are indicated by a larger Nt value. Therefore, the temperature difference across the boundary layer tends to increase as Nt values climb. Furthermore, as Nt determines the concentration of nanoparticles, higher Nt values have a substantial impact on it. The correlation between the concentration of nanofluid models and chemical reaction ($\sigma$) is shown in Fig. 3f. This suggests that the concentration of nanofluid models decreases as ($\sigma$) grows.

Tables 2, 3, and 4 show the influence of various regulating amounts on the skin friction number, Nusselt number, and Sherwood number for Casson, Williamson, and Prandlt nanofluids. It is analyzed that as *M* and $\alpha$ increase in value, the skin friction number grows as shown in Table 2. It is observed from Table 3, the Nusselt number falls as inputs of *M*, *Rd*, *Nt*, and *Nb* rise for Casson, Williamson, and Prandlt nanofluid models. The Sherwood number of Casson, Williamson, and Prandlt nanofluids has decreasing trend for higher inputs of *M*, *Nt*, *E* and increasing behavior for larger inputs of *Nb* as shown in Table 4.

**Table 2:** Numerical outcomes of skin friction coefficient for various inputs of parameters.

*M*	$\alpha$	Skin friction	CPU time (s)	Skin friction	CPU time (s)	Skin friction	CPU time (s)
		Casson case		Williamson case		Prandlt case	
1.2	30.0	1.2989	0.000242	0.9810	0.001228	0.9435	0.000224
3.2		1.798	0.000207	0.9915	0.000267	1.1607	0.000174
5.2		2.299	0.000127	1.6788	0.000373	1.3643	0.000219
1.0	45.0	1.4908	0.000165	0.9573	0.000292	− 1.0324	0.000276
	60.0	1.7535	0.000225	0.9769	0.000175	− 1.1397	0.000273
	75.0	1.9330	0.000203	0.9988	0.000193	− 1.2159	0.000249

**Table 3:** Numerical outcomes of Nusselt number for various inputs of parameters.

*M*	*Rd*	*Nt*	*Nb*	Nusselt number	Nusselt number	Nusselt number
				Casson case	Williamson case	Prandlt case
1.2	0.2	0.1	0.2	0.2811	0.2596	0.2533
3.2				0.2768	0.2522	0.2479
5.2				0.2732	0.2504	0.2440
1.0	0.5			0.3858	0.3541	0.3445
	1.0			0.5013	0.4569	0.4439
	1.5			0.6044	0.5485	0.5324
	0.2	0.2		0.2804	0.2592	0.2527
		0.5		0.2766	0.2554	0.2489
		0.8		0.2728	0.2516	0.2451
		0.1	0.3	0.2816	0.2605	0.2539
			0.6	0.2698	0.2492	0.2429
			0.9	0.2578	0.2378	0.2318

**Table 4:** Numerical outcomes of Sherwood number for various inputs of parameters.

				Sherwood number	Sherwood number	Sherwood number
*M*	*Nt*	*Nb*	*E*	Casson case	Williamson case	Prandlt case
1.2	0.1	0.2	0.5	0.4943	0.4351	0.4203
3.2				0.4806	0.4186	0.4091
5.2				0.4699	0.4144	0.4014
1.0	0.2			0.4320	0.3770	0.3630
	0.5			0.2475	0.2034	0.1937
	0.8			0.0740	0.0406	0.0351
	0.1	0.3		0.5191	0.4590	0.4431
		0.6		0.5424	0.4811	0.4646
		0.9		0.5504	0.4886	0.4720
		0.2	0.7	0.4420	0.3693	0.3491
			1.3	0.3999	0.3118	0.2856
			1.9	0.3745	0.2742	0.2424

## Conclusion

The increase of heat distribution for the MHD flow of Casson, Williamson, and Prandtl nanofluids along a stretching sheet is investigated using the Bvp4C technique. The numerical results are computed for velocity components, temperature, concentration profiles, skin friction coefficients, Nusselt and Sherwood numbers for these non-Newtonian fluids. The impacts of different regulating factors on the flow analysis are examined in the following sections through the presentation of several diagrams and a table with numerical data. The validity of the existing findings is further demonstrated by the addition of a comparison table. The following represent a few of the important findings.

- The velocity curves of Casson, Williamson, and Prandtl nanofluids exhibit a decreasing tendency as the magnetic parameter and angle of inclination is increased.
- Also, the Casson nanofluid has a low velocity as compared to the Williamson, and Prandtl nanofluids.
- The temperature curves of Casson, Williamson, and Prandtl nanofluids increase when the magnetic parameter, thermal Biot number, thermal radiation, Brownian motion, and thermophoresis increase.
- The heat flux of Prandtl nanofluid improved faster than that of Casson and Williamson nanofluid.
- Due to augmented Lewis number and Brownian motion parameters, the concentration profiles of Casson, Williamson, and Prandtl nanofluids have a decreased attribute.
- With increasing magnetic, activation energy, and thermophoresis parameters concentration curves of Casson, Williamson, and Prandtl nanofluids rise.
- The concentration of Casson nanofluid enhanced than Prandtl and Williamson nanofluid in magnetic, activation energy, and thermophoresis parameters and low in Lewis number and Brownian motion parameters.
- The skin friction of Casson, Williamson, and Prandtl nanofluids increased with the incremental inclined magnetic parameter.
- The Nusselt and Sherwood numbers declined with the increase of magnetic and thermophoresis parameters for Casson, Williamson, and Prandtl nanofluids.

Non-Newtonian nanofluid models applied over stretching sheets are applicable in a variety of real-world circumstances, particularly when taking into consideration aspects such as linear radiation and activation energy effects. These models are essential for improving drug delivery systems in biomedical engineering, heat exchanger optimisation for HVAC systems and thermal power plants, metal forming and polymer processing manufacturing processes, and oil and gas industry operations such as enhanced oil recovery and drilling fluids. In addition, they find use in renewable energy technologies such as solar thermal systems and geothermal energy, and they support environmental engineering initiatives by helping with groundwater remediation and wastewater treatment. All things considered, these models propel progress in a variety of industries, enhancing productivity, long-term viability and advances in technology.

## Future direction

This work can be extended for hybrid and ternary nanoparticles. Also, this work can be explored further for the Modified Buongiorno model.

## Data Availability

The data that support the findings of this study are available from the corresponding author upon reasonable request.
